# Synthesis of Zinc Oxide Nanoparticles by Ecofriendly Routes: Adsorbent for Copper Removal From Wastewater

**DOI:** 10.3389/fchem.2020.571790

**Published:** 2020-11-27

**Authors:** Julia de O. Primo, Carla Bittencourt, Selene Acosta, Ayrton Sierra-Castillo, Jean-François Colomer, Silvia Jaerger, Verônica C. Teixeira, Fauze J. Anaissi

**Affiliations:** ^1^Laboratório de Materiais e Compostos Inorgânicos (LabMat), Departamento de Química, Universidade Estadual Do Centro-Oeste, Guarapuava, Brazil; ^2^Chimie des Interactions Plasma-Surface (ChIPS), Research Institute for Materials Science and Engineering, Université de Mons, Mons, Belgium; ^3^Research Group on Carbon Nanostructures (CARBONNAGe), Université de Namur, Namur, Belgium; ^4^Laboratório Nacional de Luz Síncrotron (LNLS), Centro Nacional de Pesquisa em Energia e Materiais (CNPEM), Campinas, Brazil

**Keywords:** zinc oxide, starch, aloe vera, copper ion, water treatment

## Abstract

Zinc Oxide nanoparticles have been synthesized by two simple routes using Aloe vera (green synthesis, route I) or Cassava starch (gelatinization, route II). The XRD patterns and Raman spectra show that both synthesis routes lead to single-phase ZnO. XPS results indicate the presence of zinc atoms with oxidation state Zn^2+^. SEM images of the ZnO nanoparticles synthesized using Cassava starch show the presence of pseudo-spherical nanoparticles and nanosheets, while just pseudo-spherical nanoparticles were observed when Aloe vera was used. The UV-Vis spectra showed a slight difference in the absorption edge of the ZnO particles obtained using Aloe vera (3.18 eV) and Cassava starch (3.24 eV). The ZnO nanoparticles were tested as adsorbents for the removal of copper in wastewater, it is shown that at low Cu^2+^ ion concentration (~40 mg/L) the nanoparticles synthesized by both routes have the same removal efficiency, however, increasing the absorbate concentration (> 80 mg/L) the ZnO nanoparticles synthesized using Aloe vera have a higher removal efficiency. The synthesized ZnO nanoparticles can be used as effective and environmental-friendly metal trace absorbers in wastewater.

## Introduction

The fast growth of the human population and the further development of industries have direct consequences on the environment, leading to the depletion of natural resources, with an emphasis on freshwater resources. The disposal of industrial, agricultural and domestic waste often contains heavy metals that are toxic to humans and other living species with long-term intake. Among these, copper is one of the most abundant pollutants in wastewater (Ali et al., 2016), widely used in electroplating industries (Rafiq et al., 2014), welding processes, agricultural processes, plumbing material, and electrical wiring (Ali et al., 2016), its high consumption results in the presence of high amounts of this element in wasterwater. The toxic effects of this heavy metal, caused by bioaccumulation, can cause lung cancer, brain, liver, and kidney health problems, among others (Aksu and Işoǧlu, 2005; Saleh, 2017). Therefore, it is crucial for the protection of the environment and for human health to remove this metal from industrial wasterwaters before it is disposed of.

Different techniques for the removal of copper from wastewaters have being proposed (Fu and Wang, 2011), among them we can cite precipitation (Negrea et al., 2008), electrocoagulation (Dermentzis et al., 2011), filtration (Kebria et al., 2015) and ion exchange (Da̧browski et al., 2004). However, most of these methods are expensive and prove ineffective in removing heavy metals in trace concentrations. In this context, the adsorption method has stood out, due to its low-cost, ease of use (Pan et al., 2003; Rafiq et al., 2014; Ali et al., 2016) and the possibility of recycling the adsorbent. Considering the importance of treating wastewater with ion removal at trace levels, in this work, ZnO (Zinc oxide) nanoparticles were used as an adsorbent. ZnO nanoparticles have been reported as good adsorbent of positive metal ions in wastewater (Singh et al., 2011; Wang et al., 2013a). The Zinc oxide is a type-n material belonging to the semiconductor group of II-VI, has a band-gap of 3.37 eV. It is one of the most widely studied oxides due to its singular physicochemical properties, that include high chemical stability, and wide light absorption range. Zinc oxide has been announced as active material in a myriad of applications such as antifungal (Kavyashree et al., 2015; Sharma and Ghose, 2015), drug delivery (Yuan et al., 2010; Chen et al., 2013), antibacterial (Jones et al., 2008; Applerot et al., 2009), photocatalysts (Banerjee et al., 2012; Lee et al., 2016), gas sensors (Rai and Yu, 2012; Waclawik et al., 2012) and antioxidant (Kumar et al., 2014). Due to its interesting properties and high applicability, various techniques have been reported for the ZnO synthesis (Kolodziejczak-Radzimska and Jesionowski, 2014).

In this work, in addition to the use of ZnO nanoparticles as a copper ion adsorbent, it is described two low-toxicity routes to synthesize ZnO particles, which are easy to reproduce. The route I uses Aloe vera as an additive while route II uses Cassava starch. The use of these natural additives makes the synthesis more environmentally friendly, due to their high chemical reactivity and high combustion power, reducing the calcination temperature often used in the synthesis of the oxide, in addition, to act as complexing gelling. Aloe vera (Aloe barbadensis Miller) is a perennial plant belonging to the Liliaceae family, it consists mainly of glycoproteins, anthraquinones, saccharides, and others low-molecular-weight substances (Choi and Chung, 2003); inside the leaves, there is a mucilaginous gel produced by the parenchymatous cells. Cassava starch, however, is a polysaccharide of biological, non-toxic, inexhaustible biocompatible, and biodegradable source (Visinescu et al., 2011). The use of Starch and Aloe vera in the synthesis of ZnO nanoparticles has been reported (Sangeetha et al., 2011; Khorsand Zak et al., 2013; Thirumavalavan et al., 2013; Carp et al., 2015; Kavyashree et al., 2015), however, here we propose simple routes with fewer steps for the synthesis of ZnO nanoparticles.

## Experimental

### Materials

All the chemicals used were of analytical grade. Zinc nitrate hexahydrate (Zn(NO_3_)_2_·6H_2_O, 98%) was purchased from Dynamic and copper nitrate trihydrate (Cu(NO_3_)_2_·3H_2_O, 99%) was purchased from Vetec (Sigma-Aldrich). All solutions were prepared with deionized water. Natural Cassava starch in the form of colloidal suspension was used as fuel. Aloe vera leaves were harvested in the São José region of Parana-Brazil. To obtain the extract of Aloe gel, about 200 g of Aloe vera leaves were washed with deionized water and the internal mucilaginous gel was extracted. Afterward, the mucilaginous gel was crushed using a pistil and a ceramic mortar to obtain the complete extract. Finally, the solution was washed, filtered and the resulting Aloe vera gel broth extract was stored under refrigeration (2°C).

### Synthesis of Zinc Oxide

Two different routes, both easy to reproduce, were used for synthesizing Zinc oxide nanoparticles. In the route I (green synthesis), adapted from Sangeetha et al. (2011), Aloe vera (AL) gel broth extracts at the concentration (90%) were prepared with distilled water, the volume was made up to 100 ml. Subsequently, zinc nitrate (9.40 g) was dissolved in the aloe extract solution under constant magnetic stirring (120 min.) and left at rest for 12 h. The suspension was calcined in a muffle furnace at temperatures (750 °C) for 1 h. In the route II (gelatinization method): first starch (ST) was extracted from 100 g of natural Cassava starch in 300 ml of distilled water under mechanical stirring for 2 h. It was then sieved, and in the colloidal starch suspension was added 9.40 g of zinc nitrate. After 60 min of mechanical stirring (600 rpm), the suspension was calcined in a muffle furnace at a temperature of 750 °C for 1 h (Primo et al., 2019). The ZnO nanoparticles obtained were named Zn-AL (route I) and Zn-ST (route II).

### Characterization Techniques

X-ray powder diffraction profile was performed at the Brazilian Synchrotron Light Laboratory (LNLS, using XRD1 beamline, 12 keV energy, λ = 1.033 Å, 2θ of 0°-80°) (Carvalho et al., 2016). Scanning electron microscopy images were recorded using a JEOL-JSM-7500F Field Emission Scanning Electron Microscope operated 15 kV, the spatial resolution was 2.5 nm. The Raman spectra were recorded using a Micro-Raman system, Senterra Bruker Optik GmbH), λ = 532 nm, laser power 5 mW, time 10 s, resolution 4 cm^−1^. The optical diffuse reflectance was measured (UV-VIS-NIR Spectrophotometer CARYb5G, Varian) in the range of 300–800 nm. Zeta potential was recorded using ZETASIZER NANO ZS90 (MALVERN), model ZEN 1,010 at 25°C. The zeta potentials of the nanoparticles were determined from their electrophoretic mobilities according to Smoluchowski's equation (O'Brien and Hunter, 1981); the pH of these nanoparticles was adjusted between 3 and 11 using HCl or NaOH solutions. The chemical composition was evaluated by X-ray photoelectron spectroscopy (XPS) (Versaprobe PHI 5,000 from Physical Electronics, equipped with a monochromatic Al Kα X-ray source). The XPS spectra were collected at a take-off angle of 45° with respect to the electron energy analyzer and the spot size was 200 μm. Pass energy (PE) of 20 eV was used for the high-energy resolution spectra (Zn 2p, O 1s, and C 1s). The spectra were analyzed using the CASA-XPS software.

The metal ion solutions were analyzed using a Varian^TM^ SpectrAA® 220 Flame Atomic Absorption Spectrometer (FAAS). The FAAS was equipped with an air-acetylene burner. The hollow cathode lamp was set at 4 mA and the analytical wavelength was adjusted at 324.8 nm. The slit size was adjusted to 0.2 nm. The standard curve was drawn by using copper standard solutions. After the adsorption, the ZnO nanoparticles were characterized concerning their composition by energy dispersive X-ray spectrometer (EDX) from Shimadzu, model EDX-7000, containing a Rh tube, operating at 50 and 15 W. The crystalline phases were identified by powder X-ray diffraction (XRD) performed on a Bruker model D2 Phaser with Cu Kα radiation (λ = 1.5418 Å), with scan in 2θ from 10° to 90° and step rate of 0.2°/s. The zeta potential was recorded using ZETASIZER NANO ZS90 from MALVERN, model ZEN 1010. The electronic spectra of the powdered pigments samples were measured on the range of 400–900 nm with a UV-Vis Ocean Optics spectrophotometer model USB-2000.

### Adsorption Measurements

To investigate the efficiency of the ZnO nanoparticles as adsorbents for the removal of copper metal ions from water, an adsorption test was performed. The parameters: contact time; initial pH and initial metal ion concentration were investigated. The adsorption experiments were carried out in conical flasks containing 25 mL of copper solution with an initial concentration ranging from 40 to 120 mg L^−1^. To this end, 250 mg of the ZnO particles were added, and the solutions were kept under continuous shaking for 240 min in a heating bath at 25°C. To study the adsorption kinetics and the pH parameters, 50 mg L^−1^ of a solution containing Cu (II) and the same amount of ZnO particles was prepared; its pH was adjusted using 0.1 HCl and 0.1 NaOH solutions. The resulting solutions were centrifuged at 1,200 rpm for 15 min. The ion concentration measurements were performed before the adsorption test without the presence of the adsorbent and after 4 h of adsorption in a flame atomic absorption spectrometer (FAAS).

The amount of Cu^2+^ ion adsorbed at the end of the adsorption experiment and the ion percentage removal (%) by the ZnO nanoparticles were calculated applying Equations (1, 2), respectively:

(1) $$\begin{array}{r} q=\frac{\left(Co-Cf\right)}{m}.V \end{array}$$

(2) $$\begin{array}{r} \%Removal=100.\frac{\left(Co-Cf\right)}{Co} \end{array}$$

where *q* is the amount of ion adsorbed by the adsorbent in mg g^−1^, *C*_*o*_ is the initial ion concentration in contact with the adsorbent (mg.L^−1^), *C*_*f*_ is the ion concentration (mg.L^−1^) after the batch adsorption process, *m* (g) is the mass of adsorbent and V (L) is the volume of ion solution.

### Test Leaching of Nanoparticles

To check the stability of the nanoparticles a method adapted from (Rafiq et al., 2014) was used. Thus, 50 mL of simulated sample was treated separately with 250 mg of ZnO synthesized. The initial pH of the experiment was 4 or 6 and the contents were allowed to remain in contact for 240 min while maintaining the temperature at 25°C. After centrifugation and filtration, the residue was washed with deionized water followed by oven drying at 60°C.

## Results and Discussion

### Characterization of the Zinc Oxides Nanoparticles

**Figure 1:**
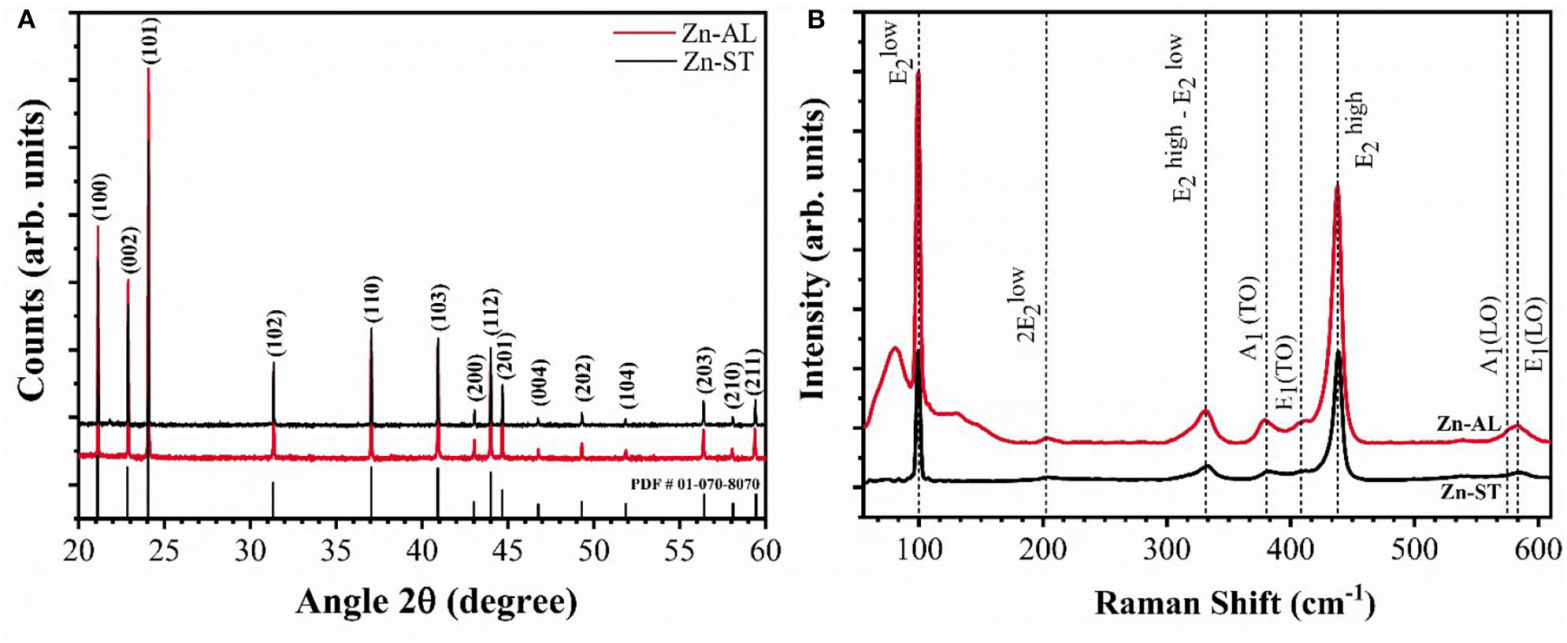
**(A)** XRD pattern and **(B)** Raman spectrum of the ZnO samples.

Figure 1A presents the X-ray diffractograms of the zinc oxides nanoparticles obtained after heat treatment at 750°C for 60 min. The crystalline phase is identified by the presence of the characteristic peaks of the Wurtzite ZnO phase (Kisi and Elcombe, 1989), belonging to the compact hexagonal system with a space group P63mc to the crystallographic chart [JCPDS, #PDF01-070-8070]. Additional peaks were not detected, evidencing that the single-phase ZnO was successfully obtained regardless of the synthesis route used and the precursors were completely decomposed. The XRD patterns allowed to determine the average crystallite size of the ZnO nanoparticles, estimated by Scherrer's equation {D = 0.9λ/(B cosθ)} (Hedayati et al., 2015), with the average size of 43.3 nm for Zn-AL and 44.9 nm for Zn-ST. According to these results, the crystalline size is affected by the polysaccharide used in the synthesis, at the same calcination temperature.

Figure 1B shows the Raman spectra of the samples indicating the characteristic wurtzite phase peaks, corroborating with the XRD patterns. The predominant bands are at 99 cm^−1^ (mode E_2_low) and 437 cm^−1^ (mode E_2_high). The E_2_low mode is attributed to the vibrations of zinc sublattice in ZnO and E_2_high mode is assigned to the oxygen vibration (Cuscó et al., 2007; Stanković et al., 2012), the strong E_2_high mode indicates the high crystallinity of the oxide (Jothilakshmi et al., 2009), the same vibrational mode has been identified for the zinc oxide nanoparticles obtained via the Starch-assisted synthetic route, reported by Carp et al. (2015). The bands at 380 and 408 cm^−1^ correspond to the first-order optical modes A_1_(TO) and E_1_(TO), bands at 202 and 330 cm^−1^ are characteristic of second-order modes 2E_2_low and E_2_high—E_2_low, caused by multiphonon processes. The bands located at 573 and 584 cm-1 are assigned to A1(LO) and E1(LO) modes, these bands are associated to the presence of structural defects in the ZnO structure, being the E_1_(LO) mode strongly affected (Cuscó et al., 2007).

**Figure 2:**
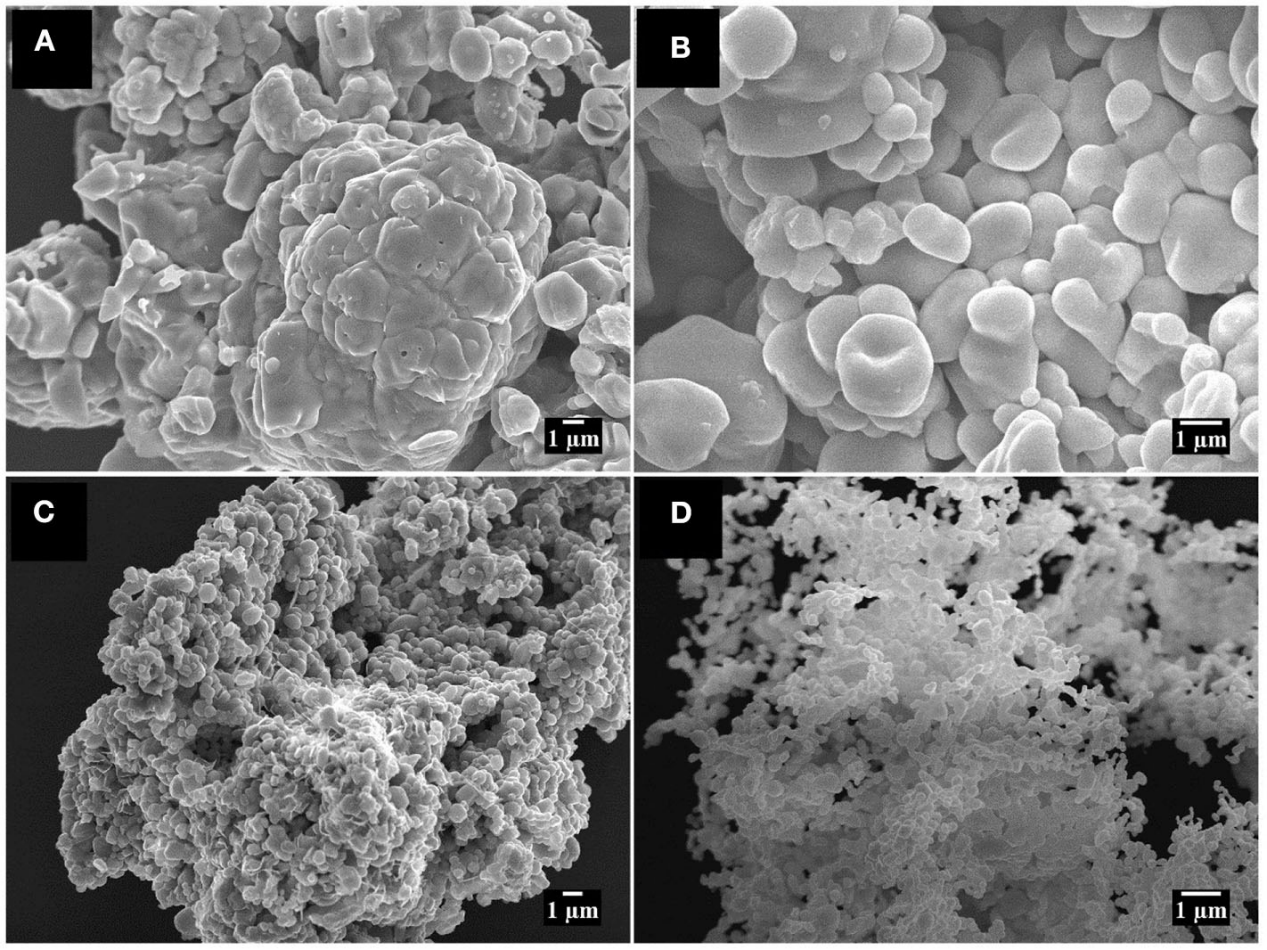
SEM image **(A)** 4,000x **(B)** 8,000x Zn-AL; and **(C)** 4,000x **(D)** 8,000x Zn-ST.

Figures 2A,B shows the SEM images for Zn-AL, which consists of pseudo-spheres, and non-uniform hexagonal particles. For Zn-ST, uniform spherical particles are formed (Figures 2C,D). The two samples show particle aggregation, related to the self-assembly effect (Khorsand Zak et al., 2013). The Zn-AL particles tend to agglomerate in plaques (Figure 2A), this was attributed to the Aloe vera gel acting as a sacrifice complexant in the formation of the ZnO nanoparticles during the combustion (Kavyashree et al., 2015). The two synthesis routes (Aloe vera and Cassava starch) have polysaccharides as fuel for the formation of ZnO nanoparticles; their formation mechanism can be described by the “egg-box” model (Kavyashree et al., 2015). Therefore, their difference in morphology can be associated with the complex polymeric network of each polysaccharide. Aloe vera gel consists of a combination of organic chains, such as soluble polysaccharides, monosaccharides, proteins, amino acids, among others (Choi and Chung, 2003). The colloidal suspension of Cassava starch is more homogeneous and less complex because it consists basically of amylopectin and amylose leading to the formation of uniform particles, since the Zn (II) ions occupy the “egg-box” more efficiently, with more regular distance. The Aloe vera gel presents a greater variation in its natural components than Cassava starch, affecting directly the shape and reproducibility of ZnO nanoparticles.

**Figure 3:**
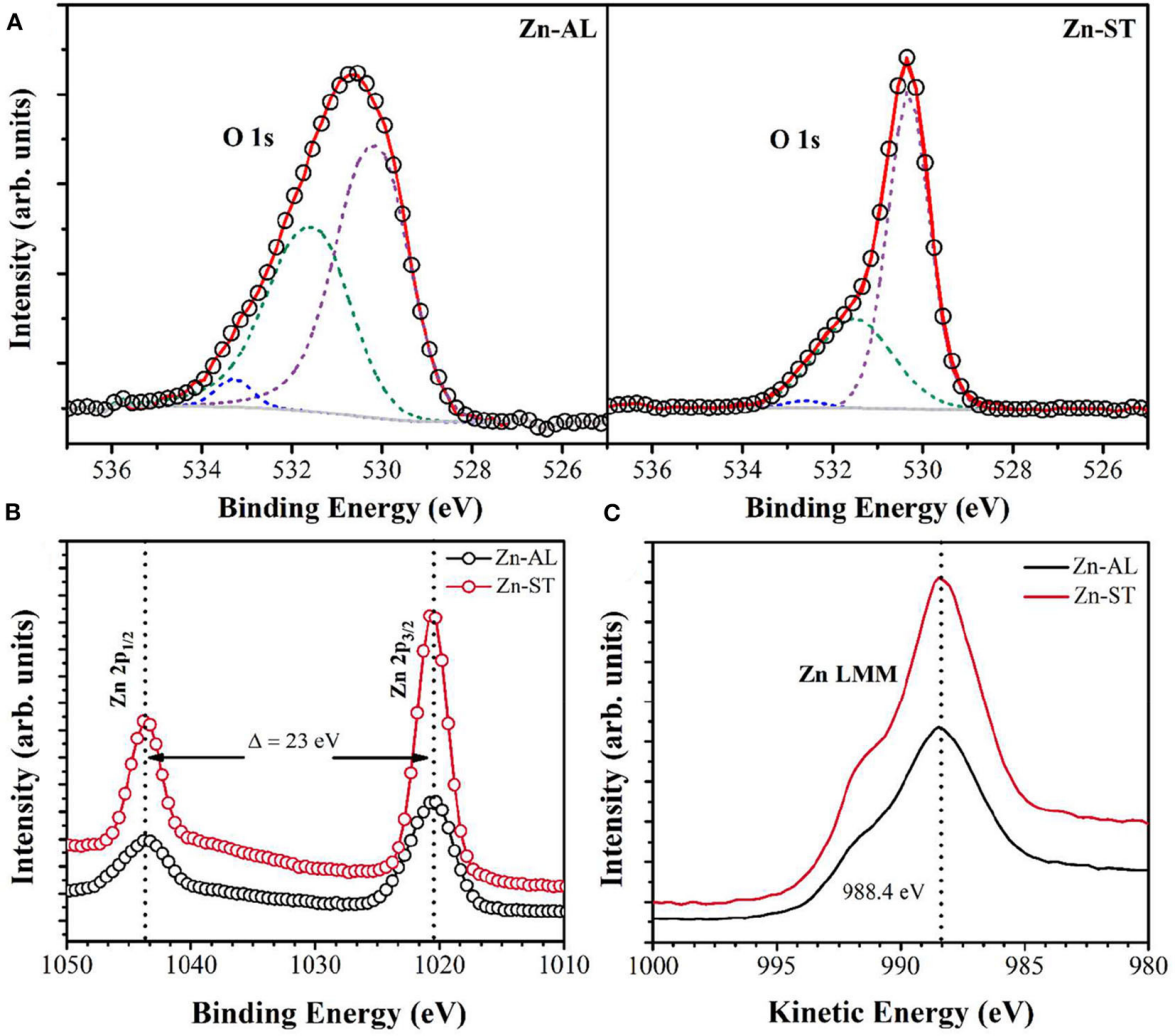
XPS spectra of **(A)** O 1s. **(B)** Zn 2p and **(C)** Zn LMM Auger of the ZnO samples.

The chemical environment of the zinc and oxygen atoms were analyzed using X-ray photoelectron spectroscopy (XPS). The O 1s and Zn 2p XPS core-level spectra are shown in Figure 3. The binding energy of the XPS data was calibrated using the C 1s peak at 284.6 eV (Das et al., 2010). The O 1s spectrum was fitted with three components centered on 530.2 ± 0.1, 531.4 ± 0.6, and 532.3 ± 0.7 eV, for both samples (Figure 3A). The low binding energy component located at 530.2 ± 0.1 eV is attributed to O^2−^ ions participating in the Zn-O bond in the wurtzite structure of the hexagonal Zn^2+^ ions of ZnO (Chen et al., 2000; Al-Gaashani et al., 2013). The component centered at 531.4 ± 0.6 is associated with photoelectrons emitted from O^2−^ ions in oxygen-deficient regions in the matrix of ZnO (Chen et al., 2000). The high binding energy component located at 532.7 ± 0.7 is reported to be associated with oxygen species adsorbed on the surface of the ZnO, such a -CO_3_, H_2_O, or O_2_ (Sangeetha et al., 2011; Visinescu et al., 2011). The Zn 2p high-resolution XPS spectra show the 2p doublet (Figure 3B) with components centered at 1020.6 eV (Zn 2p_3/2_) and 1043.5 eV (Zn 2p_1/2_). For both samples, the binding energy difference between these core levels is 23.0 eV, reference value denoting the presence of zinc in Zn^2+^ oxidation state (Chen et al., 2000; Das et al., 2010), the chemical state is confirmed by the Zn LMM Auger data (Figure 3C).

**Figure 4:**
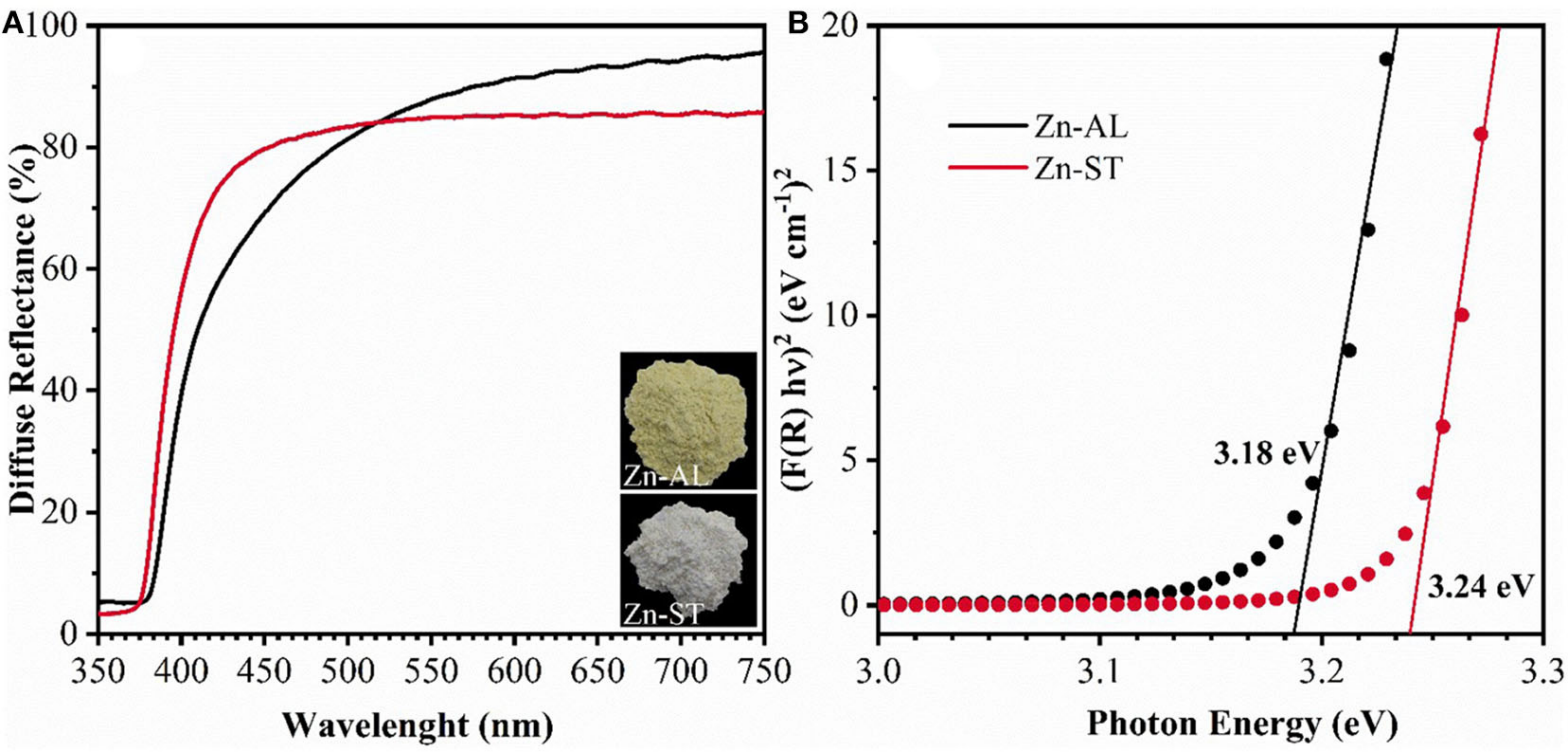
**(A)** Diffuse reflectance spectra and **(B)** Kubelka-Munk curves of ZnO samples.

Figure 4 shows the optical characterization of the ZnO nanoparticles synthesized using Aloe vera (Route I) and Cassava starch (Route II). It can be observed in Figure 4A an increase in the reflectance at wavelengths larger than 380 nm, this can be attributed to the direct band-gap of ZnO due to the electron transitions from the valence band to the conduction band (O_2p_ Zn_3d_) (Kavyashree et al., 2015), with a lower percentage of reflectance for Zn-AL (~65%). The band energy gaps (E_GAP_) of the samples were calculated using the Kubelka-Munk method (Cuscó et al., 2007), the E_GAP_ were determined by linear extrapolation of the curve [F(R) x E]2 vs. energy (E) in (Figure 4B), with values: 3.24 eV (Zn-ST) and 3.18 eV (Zn-AL), similar values have been reported in (Khorsand Zak et al., 2013; Carp et al., 2015) for zinc oxides obtained with Starch. The variation in the optical gap of the ZnO nanoparticles can be associated with a variation in the average particle size and morphology. The synthesized ZnO nanoparticles exhibit a slight red shift in the absorption edge (Figure 4A), this increase in the response range toward the visible radiation region can be explored in the future as photocatalysts with visible light activity (Stanković et al., 2012).

### Copper Ion Removal by ZnO Particles

#### Zeta Potencial (ζ) vs. pH

**Figure 5:**
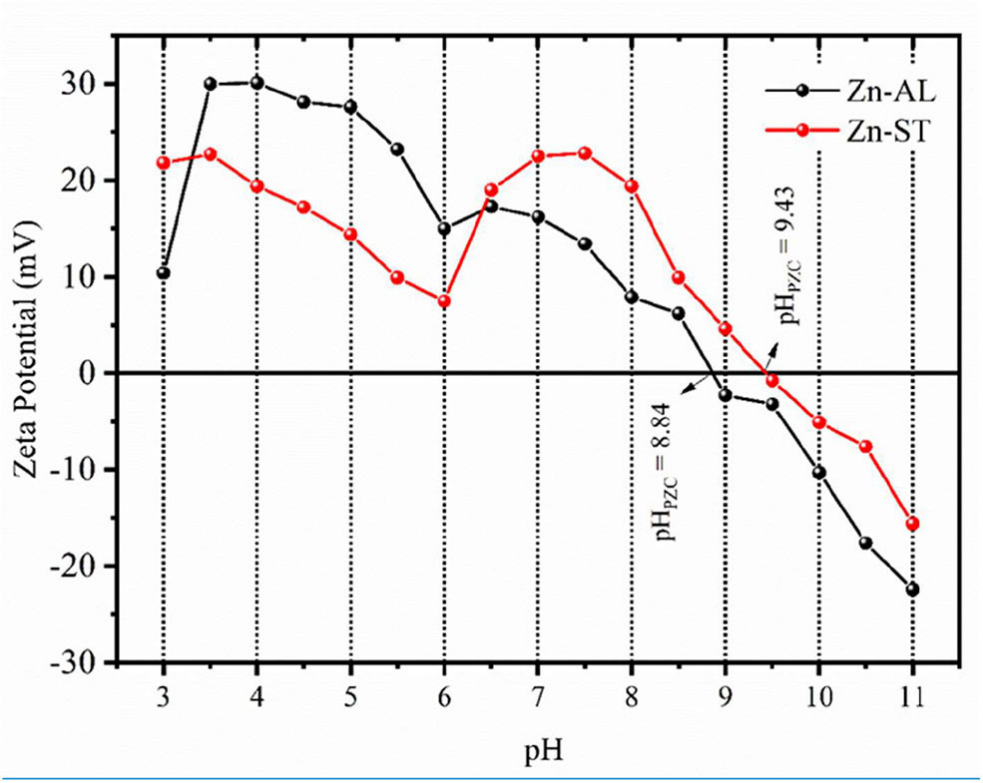
Zeta potential as a function of pH and point of zero charge (pH_PZC_) for the Zn-AL and Zn-ST samples.

Figure 5 shows the obtained ζ-potential values as a function of pH for Zn-AL and Zn-ST. The zeta potential allows evaluating if the particles in the colloidal state show chemical stability. A high ζ-potential generates electrostatic repulsion, preventing particles flocculation and aggregation (Rodrigues et al., 2020); this range of ζ-potential is located below −30 mV or above +30 mV. When the pH <6, the ZnO surface charge shows a strongly positive ζ potential value equal to + 30 ± 2 mV for Zn-ST and +24 ± 2 mV for Zn-ST. Increasing the pH, the point of zero charge (pH_PZC_) is reached at 8.8 and 9.4 for Zn-AL and Zn-ST, respectively. These values are in accordance with the values of the literature pH_PZC_ for ZnO (Adair et al., 2001; Tso et al., 2010). By further increasing the pH ≥ pH_PZC_ the ZnO nanoparticles exhibit negative surface charge values.

#### Effect of pH on Adsorption

**Figure 6:**
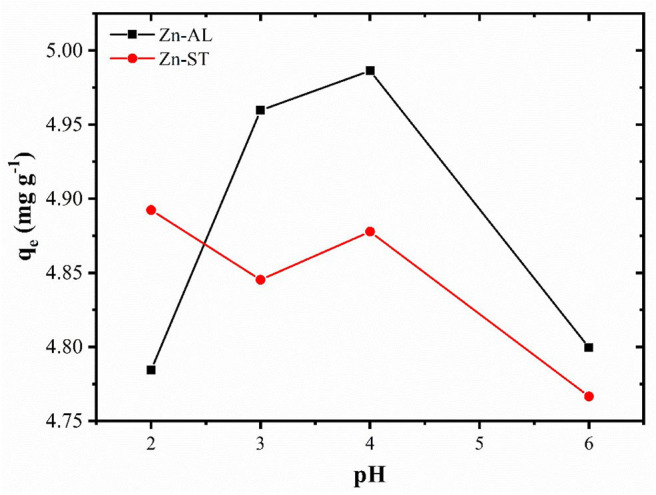
Effect of the pH on the adsorption of the Cu (II) ions by the Zn-AL and Zn-ST oxides.

In the absorption study, the pH is an important factor that affects the surface charge of the adsorbent and the degree of ionization of the ions affects the adsorption capacity. The adsorption study was carried at different pHs (2–6), because the copper ion precipitates as Cu(OH)_2_ at pH ≥ 6 (Bagheri et al., 2014). The effect of the pH in the adsorption of the heavy metal by the ZnO is shown in Figure 6. The removal of the Cu (II) ions is strongly dependent on pH, with percentage of removal > 95% for all evaluated pH, reaching the maximum adsorption at pH of 4 for Zn-AL.

The removal of Cu (II) on surface of ZnO, can be explained in terms of the adsorbent pH_PZC_ (point zero charge), for values of the pH < pH_PZC_ the adsorbent surface is protonated and positively charged, while for pH > pH_PZC_ the active sites are deprotonated and the charge is negative (Kikuchi et al., 2006; Bagheri et al., 2014). Thus, increasing the pH, the competition between H^+^ and Cu^2+^ decreases by the reduction of the repulsive force. However, in this study, the adsorption at pH < pH_PZC_ was observed (Figure 5), indicating that, the adsorption of metallic ions on the surface of ZnO may occur via non-electrostatic interaction. When the ZnO particles are exposed in water, hydroxyl groups will be formed (Wang et al., 2010; Le et al., 2019), becoming adsorptive active sites removing metal ions by reacting with OH^−^ on the ZnO surface (Bagheri et al., 2014). Thus, the mechanism of ion adsorption can be explained by the model of complexing of ion adsorption in hydrated solids (Faur-Brasquet et al., 2002; Bagheri et al., 2014), in which the Cu (II) ions interact with the active groups (OH^−^) on the oxide surface:

$$\begin{array}{l} \equiv ZnOH+Cu_{\left(aq\right)}^{2+}↔\;\equiv ZnOCu^{+\;}+\;H_{\left(aq\right)}^{+} \end{array}$$

Therefore, with an increase in the pH of the solution, the amount of active sites on the ZnO surface increases, becoming more favorable to the adsorption of the metallic ion, thus resulting in a greater removal efficiency. The decrease in the removal of Cu (II) ions at pH 6 (Figure 6), can be associated to their precipitation occurring from pH ≥ 6, and thus forming complexes that are not adsorbed by the ZnO adsorbents.

The isotherm and adsorption kinetics studies were performed without pH (pH ~ 6) adjustment. The stability of ZnO nanoparticle was checked after the adsorption experiment at pH 4 and 6. For pH4 the dried samples weight 253.2 mg and 252.8 mg, and for pH6 they weight 252.4 mg and 252.1 mg for Zn-AL and Zn-ST, respectively. The small increase in the weight compared to the initial one (250 mg) can be associated to the absence of adsorbent leaching.

#### Effect of Initial Metal Ion Concentration

**Figure 7:**
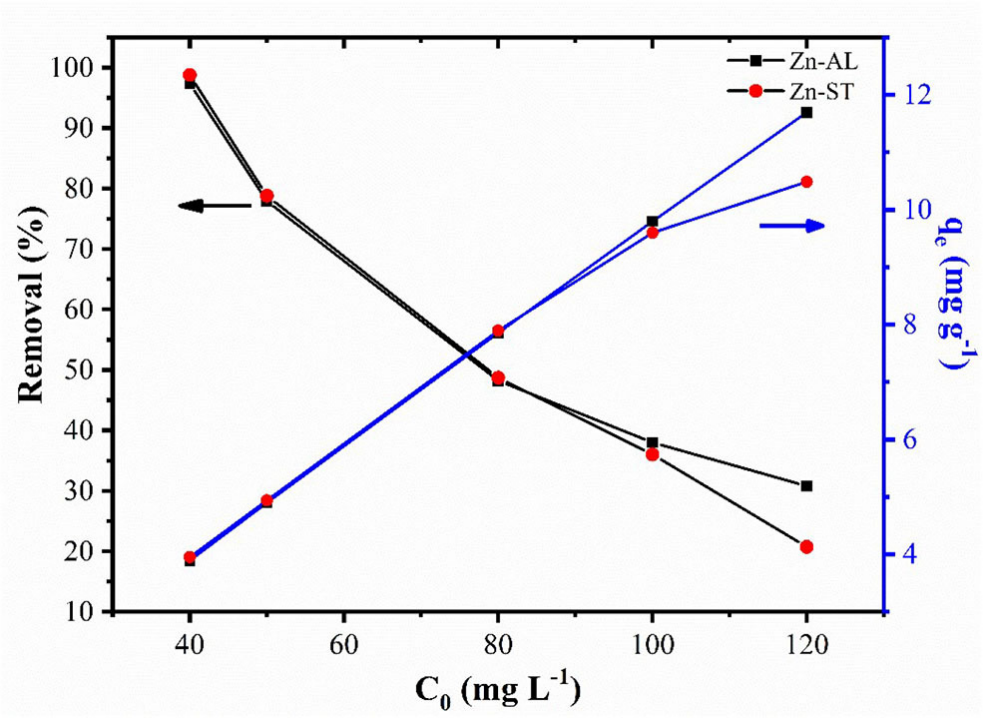
Effect of initial concentration on adsorption and equilibrium amount adsorbed of Cu (II) ion in Zn-AL and Zn-ST.

Figure 7 shows the effect of the initial metal concentration on the percentage of the Cu (II) removal. The studies were carried out at optimized contact time and temperature at 25^o^C. The results show that the percentage of removal decreases for increasing the initial concentration for both Zn-AL and Zn-ST. At low concentrations the metal ions are adsorbed by specific sites, with the increase in the concentration of Cu (II) ion occurs a saturation of these active sites, and the exchange sites are filled (Rafiq et al., 2014). Conversely, the amount of copper adsorbed per gram of adsorbent (q_e_) increased with the increase in the initial concentration of Cu ions, due to the Cu (II) ion adsorption capacity on the available adsorbent.

#### Adsorption Isotherms

**Figure 8:**
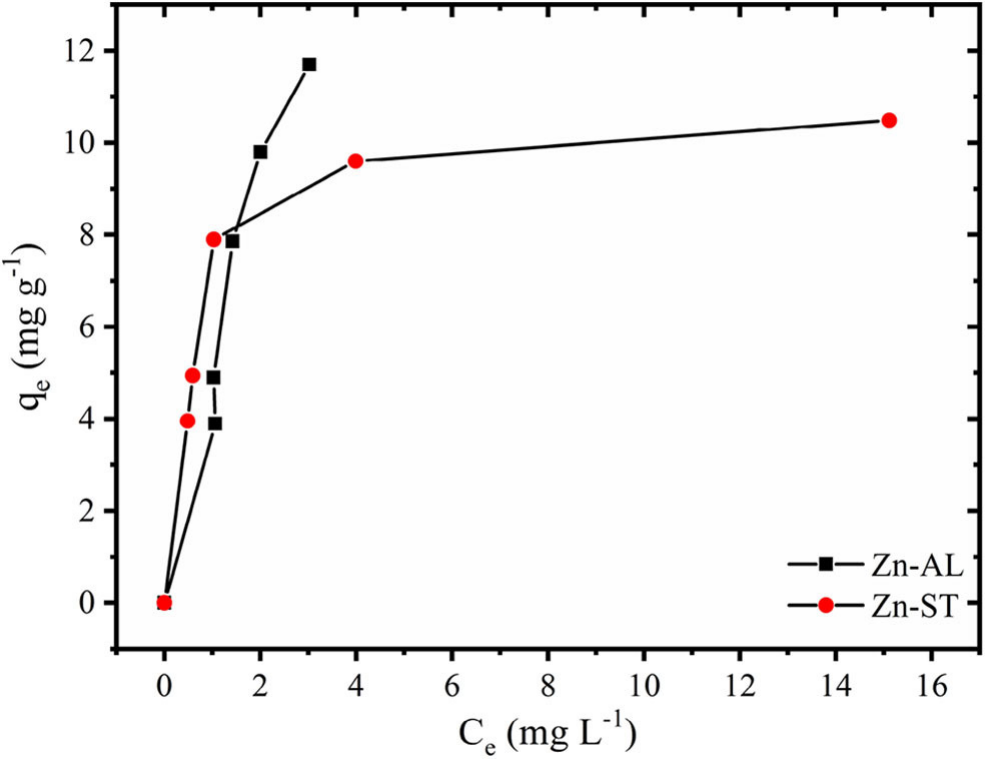
Adsorption of Cu (II) ions into ZnO samples at 25 °C.

The Cu (II) ion adsorption isotherms of Zn-AL and Zn-ST are presented in Figure 8. The results show that in the Zn-AL the saturation was not effectively reached, while the Zn-ST shows saturation when the initial concentration of 100 mg L^−1^ and 120 mg L^−1^ were investigated.

Adsorption of Cu (II) ions into Zn-AL and Zn-ST data were adjusted according to the Langmuir and the Freundlich isotherm models and their correlation parameter are presented in Table 1. The Langmuir model is applicable in systems with ideal homogeneous surface adsorption (Azizian and Bagheri, 2014; Jing et al., 2018). This isothermal model is generally defined as monolayer saturation capacity and the maximum adsorption capacity of the adsorbent for a particular adsorbate (Jaerger et al., 2015). The Freundlich model, on the other hand, reproduces better a heterogeneous system (Jaerger et al., 2015). The Langmuir isotherm in the linear form is given as (Equation 3):

(3) $$\begin{array}{r} \frac{C_{e}}{q_{e}}=\frac{1}{q-maxK_{L}}+\frac{C_{e}}{q_{\max}} \end{array}$$

where *q*_*e*_ (mg g^−1^) is the amount of ions adsorbed per unit mass of ZnO at equilibrium; *K*_*L*_ (L mg^−1^) is the Langmuir constant related to the affinity of the binding sites; *q*_*max*_ (mg g^−1^) is a parameter related to the maximum amount of Cu (II) per unit weight of ZnO.

**Table 1:** Parameters of the Langmuir isotherms and the Freundlich for adsorption of Cu (II) ions into ZnO samples.

**Sample**	**Langmuir**			**Freundlich**		
	**KL (L mg^**−1**^)**	**q_**max**_ (mg g^**−1**^)**	***r*^**2**^**	**KF (L g^**−1**^)**	**1/n**	***r*^**2**^**
Zn-AL	0.447	20.425	0.992	4.603	1.057	0.822
Zn-ST	1.562	10.952	0.999	5.939	3.868	0.705

The Freundlich isotherm is an empirical model and is commonly used for low concentrations of adsorbate (Jaerger et al., 2015). The linearized form of the Freundlich isotherm is given as (Equation 4):

(4) $$\begin{array}{r} \text{In}q_{e}=\text{In}K_{F}+\frac{1}{n}\text{In}C_{e} \end{array}$$

where *K*_*F*_ (mg L^−1^) is the Freundlich constant; *n* is a parameter related to the intensity of adsorption and the system heterogeneity. *K*_*F*_ and *n* are the Freundlich constants determined from the intercept and slope of the straight line of the plot ln*q*_*e*_ vs. ln*C*_*e*_.

The correlation coefficients (*r*^2^) obtained by the Langmuir isothermal model were well-fitted as shown in Table 1. The adsorption process consists of monolayer adsorption of Cu (II) ions at the ZnO nanoparticles surface, this is observed for the nanoparticle synthesized by both routes. Another important property obtained analyzing the Langmuir isothermal is the maximum adsorption capacity (q_max_). The values of q_max_ for Zn-AL and Zn-ST were 20.42 and 10.95 mg g^−1^, respectively. The maximum adsorption values obtained in this study are relatively low compared with data reported in the literature (Table 2) (Azizian and Bagheri, 2014; Jing et al., 2018). However, in the present study, the interest is in the removal of low concentrations, and saturation was not observed for both Zn-AL and Zn-ST as adsorbent. The ZnO nanoparticles produced by both routes have good characteristics as Cu (II) ion adsorbent showing a percentage of removal near to 98% for low metal concentration (Figure 7) indicating that the synthesized nanoparticles are potential adsorbent of metal traces in wastewater.

**Table 2:** Reported adsorption capacities (mg g^−1^) of copper using Zinc Oxide as adsorbent.

**Adsorbent**	**Adsorption capacities (mg g^−1^) (q_**max**_)**	**References**
ZnO nanoplates	> 1600.0	Wang et al., 2010
Nanocomposite of ZnO with montmorillonite	54.06	Sani et al., 2017
ZnO nanoparticles	625.0	Rafiq et al., 2014
ZnO@chitosan core-shell nanocomposite	117.6	Saad et al. (2018)
ZnO hollow microspheres	1400	Wang et al., 2013b
ZnO from Tecnan	137.5	Mahdavi et al., 2012
ZnO (flower-like)	692	Bagheri et al., 2014
Porous Zn-AL	20.43	Present work
porous Zn-ST	10.95	Present work

#### Effect of Contact Time

**Figure 9:**
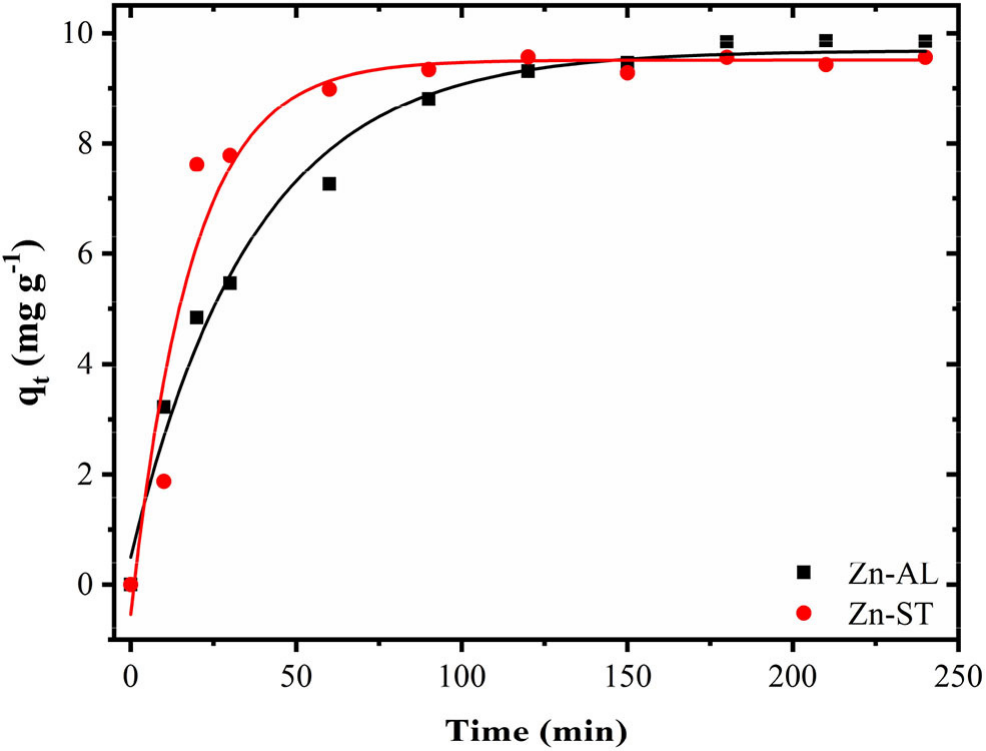
Progressive removal of Cu (II) ions from aqueous solutions using Zn-AL and Zn-ST as adsorbent.

Figure 9 shows the effect of contact time on the Cu (II) adsorption. The adsorption of copper by ZnO nanoparticles was investigated as a function of contact time in the range between 5 min and 240 min with 50 mg L^−1^ initial ZnO concentration. The value of the copper removal gradually increases with the time until the equilibrium is reached within 120 min. No significant increase occurred between 180 and 240 min until the adsorption equilibrium was reached. Both Zn-AL and Zn-ST reached the Cu (II) ion adsorption equilibrium at 150 min. The pseudo-first-order model is widely used in solute adsorption in a liquid solution and is represented by Equation (5):

(5) $$\begin{array}{r} \ln\left(q_{e}-q_{t}\right)=\ln\;q_{e}-k_{1}t \end{array}$$

where *q*_*t*_ (mg g^−1^) is the amount of Cu (II) adsorbed at time t (min) and *k*_1_ is the rate constant of the pseudo-first-order adsorption (min^−1^). The pseudo-second-order kinetics equation is based on the adsorption capacity and is represented in Equation (6):

(6) $$\begin{array}{r} \frac{t}{q_{t}}=\;\frac{1}{k_{2}\;q_{e}^{2}}+\;\frac{t}{q_{e}} \end{array}$$

where *k*_2_ (g mg^−1^ min^−1^) is the pseudo-second-order adsorption rate constant.

**Figure 10:**
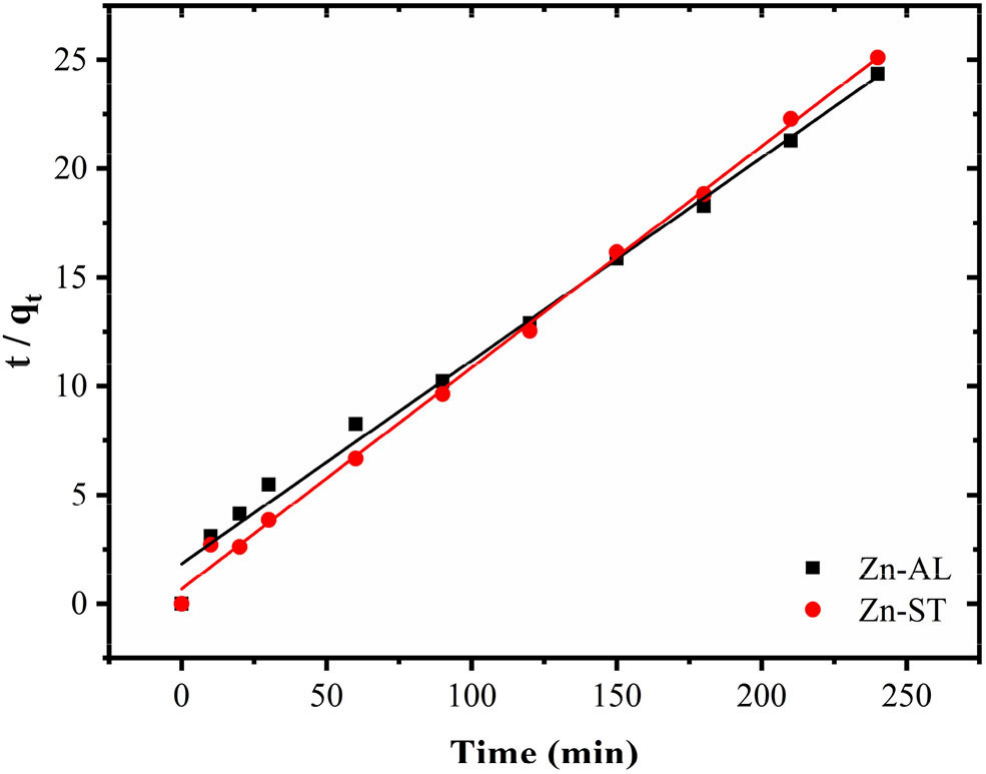
Kinetic parameters for Cu (II) removal using Zn-AL and Zn-ST as adsorbent.

Table 3 shows the values of the kinetic parameters obtained for the removal of Cu (II) ions in Zn-AL and Zn-ST. As observed (Figure 10 and Table 3), the adsorption data adjusted better to the pseudo-second-order kinetic model, since the linear correlation coefficients $r_{2}^{2}$ are above 0.99 for all Cu (II) ion solutions at 25^o^C. The *q*_*e*_ data obtained experimentally are closer to those obtained by the pseudo-second-order model, this fact indicates that the adsorption process is dependent on both the quantity of Cu (II) ions and ZnO sites available (Almeida et al., 2010; Jaerger et al., 2015). These results agreed with the report on the adsorption of metals in ZnO by Rafiq et al. (2014) and Kumar et al. (2013).

**Table 3:** Kinetic parameters for Cu (II) removal using Zn-AL and Zn-ST as adsorbent.

**Sample**	**q_**e, exp**_ (mg g^−1^)**	**Pseudo-first order**				**Pseudo-second order**		
		**C_**0**_ (mg L^−1^)**	**q_***e***_ (mg g^−1^)**	**10^**−2**^ k_**1**_ (h^−1^)**	**$r_{\text{i1}}^{2}$**	**q_**e**_ (mg g^−1^)**	**10^**−2**^ k_**2**_ (g mg^−1^h^−1^)**	**$r_{\text{i2}}^{2}$**
Zn-AL	9.87	50	6.77	2.63	0.8301	9.83	1.52	0.9973
Zn-ST	9.57	50	2.64	2.16	0.7521	9.67	2.65	0.9991

Equation (7) describes a model of the effect of the intraparticle diffusion on adsorption based on the theory proposed by Weber and Morris:

(7) $$\begin{array}{r} q_{t}=\;k_{i}t^{0.5}+C_{i} \end{array}$$

where *k*_*i*_ is the rate constant (mg g^−1^ t^−0.5^) and values of *C*_*i*_ give information regarding the thickness of the boundary layer.

**Figure 11:**
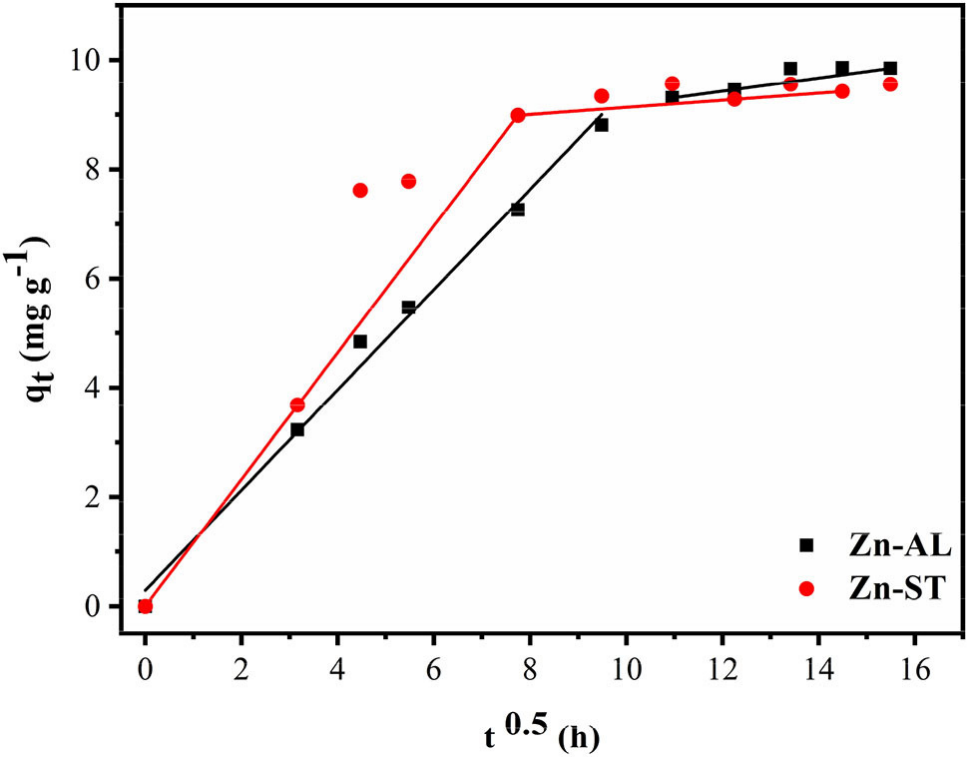
An intraparticle diffusion model for Cu (II) removal using Zn-AL and Zn-ST as adsorbent.

When the adsorption mechanism follows the intraparticle diffusion process *k*_*i*_ can be obtained from *q*_*t*_ vs. *t*^0.5^ plot. In this study, the data displayed multilinear graphs, governed by two steps as shown in Figure 11. This fact indicates that the adsorption process involves more than one mode in the adsorption of the Cu (II) ion by the ZnO nanoparticles. The linear segment of the adsorption curve is attributed to the immediate adsorption occurring at sites available on the oxide surface. While the second linear portion refers to the adsorption in the final stages of the adsorption equilibrium, where the intraparticle diffusion process begins to decrease and reach a plateau due to the low concentration of remaining Cu (II) ions or because the maximum adsorption by the adsorbate is achieved (Rafiq et al., 2014; Jaerger et al., 2015). The results of the intraparticle diffusion for Cu (II) ions in both Zn-AL and Zn-ST oxides suggest that the adsorption is controlled initially by the external mass transfer, followed by the mass transfer by the intraparticle diffusion until reaching equilibrium (Almeida et al., 2010; Jaerger et al., 2015). These steps agree with the decrease in the diffusion rate going from k_i1_> k_i2_ corroborating with the increase in the thickness of the limit layer C_i1_ < C_i2_ (Rafiq et al., 2014) as observed in Table 4.

**Table 4:** Intra-particle diffusion constants for Cu (II) removal using ZnO as adsorbent.

**Sample**	**10^−2^ k_**i1**_ (mg g^−1^ min^−0.5^)**	**C_**i1**_ (mg g^−1^)**	**$r_{\text{i1}}^{2}$**	**10^−2^ k_**i2**_ (mgg^−1^ min^−0.5^)**	**C_**i2**_ (mg g^−1^)**	**$r_{\text{i2}}^{2}$**
Zn-AL	0.918	0.292	0.9906	0.120	7.989	0.9996
Zn-ST	1.160	0.006	1.0000	0.065	8.482	1.0000

### Characterization of Cu/ZnO Nanoparticles

After the adsorption assay, the samples at pH 4 and 6 were dried at 60 °C in an oven-dry for 12 h and characterized. The chemical composition data (EDXRF) evince the incorporation of the Cu (II) ions in both Zn-AL and Zn-ST nanoparticles following their surface adsorption (Table 5).

**Table 5:** Compositional chemical analysis data by EDXRF (% element).

**Samples**	**ZnO (%)**	**Adsorption**	**ZnO (%)**	**Cu (%)**	**Estimated composition**
Zn-AL	98.4	pH 4	97.2	0.8	ZnOCu_0.008_
		pH 6	97.4	0.9	ZnOCu_0.009_
Zn-ST	98.6	pH 4	97.6	0.7	ZnOCu_0.007_
		pH 6	97.8	0.8	ZnOCu_0.008_

**Figure 12:**
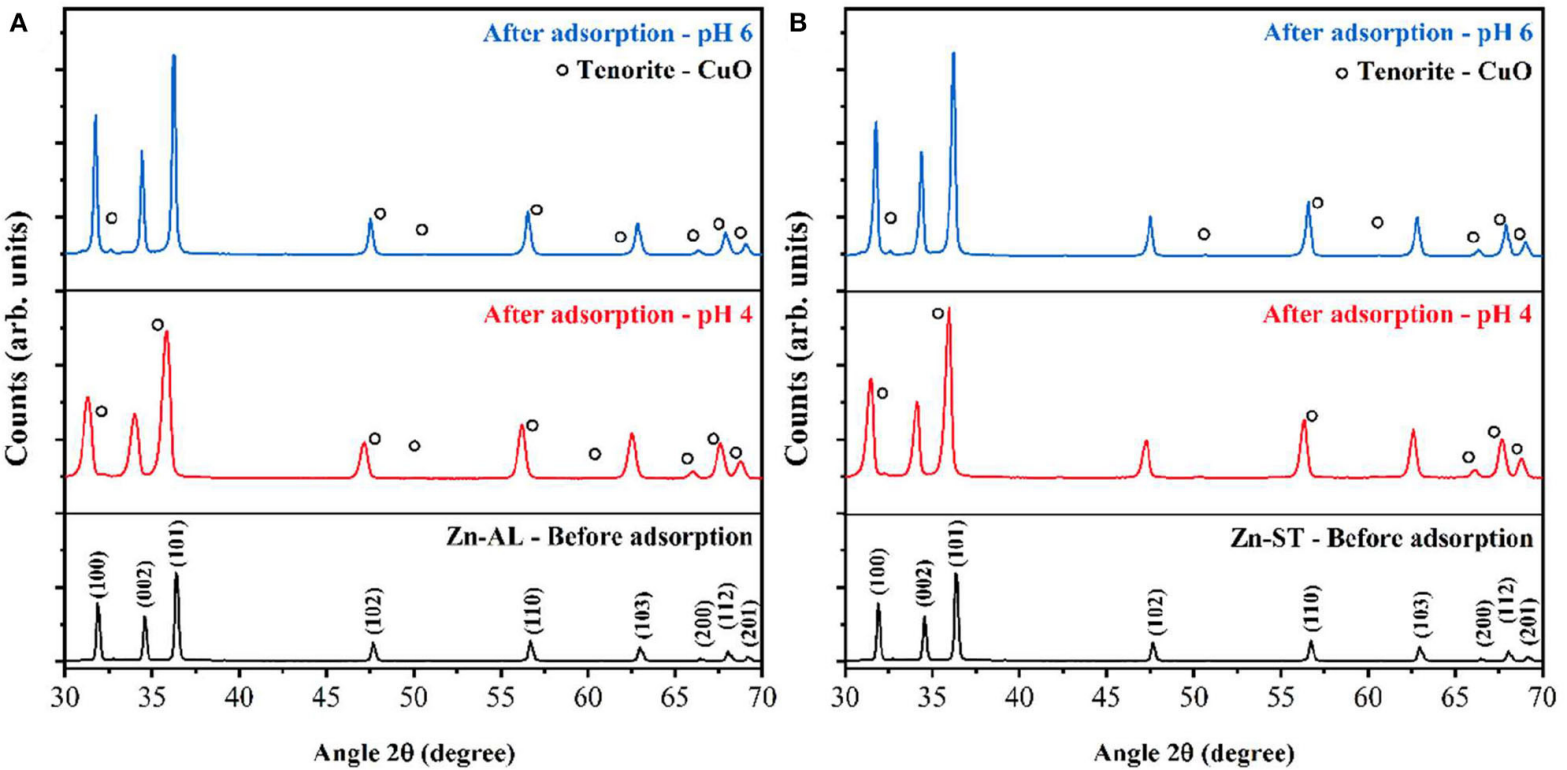
XRD pattern before adsorption of copper solution 50 mg L^−1^, pH 4, and 6: **(A)** Zn-AL and **(B)** Zn-ST.

Figure 12 shows the XRD patterns recorded on the ZnO nanoparticles before and after the Cu (II) metal ions removal. It can be seen that the diffraction peaks recorded on the samples after the adsorption correspond to the majority of peaks of the ZnO hexagonal Wurtzite crystal phase (XRD recorded on the Zn-AL and Zn-ST samples before adsorption). However, in the XRD patterns recorded after adsorption, the CuO phase can be observed in both routes (I and II) samples and for different pHs. The diffraction peaks of CuO were indexed to the monoclinic Tenorite crystal phase of CuO (JCPDS, #PDF 96-901-6327). Moreover, similar to the CuO Bragg peaks the ZnO peaks slightly shift to lower diffraction angles compared to that of the ZnO recorded before adsorption, indicating the substitution of Zn^2+^ by Cu^2+^ ions in the crystal lattice (Mukhtar et al., 2012). According to (Wang et al., 2010), the hydrated Cu (II) or ${\text{Cu(H}}_{\text{2}}{\text{O)}}_{\text{6}}^{\text{2+}}$ can react with the OH^−^ groups and form Cu-O weak bounds through a Lewis interaction, in addition, the adsorbed copper ions can partially hydrolyze leading to the formation of Cu-OH and, consequently, the formation of Cu-O-Cu on the surface of ZnO, thus denoting the Tenorite phase of CuO formed on the surface of ZnO. The crystallite sizes after the adsorption were calculated using Scherrer's equation (Hedayati et al., 2015), the obtained value were: 16.31 nm (Zn-AL, pH 4), and 36.22 nm (Zn-AL, pH 6); and 20.18 nm (Zn-ST, pH 4), and 31.89 nm (Zn-ST, pH 6).

**Figure 13:**
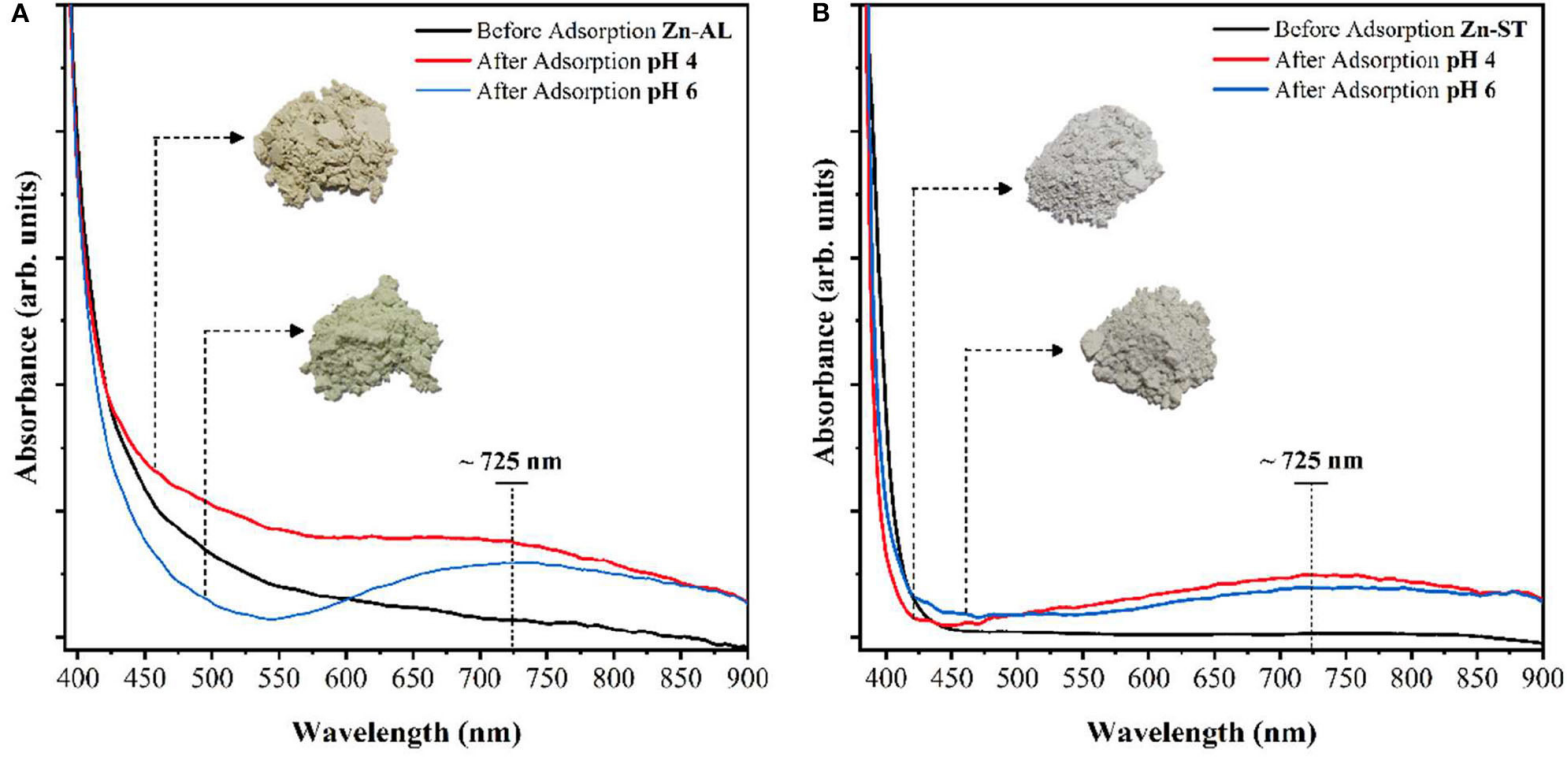
Electronic absorption spectra (visible) of ZnO before the adsorption of copper solution 50 mg L^−1^, pH 4 and 6: **(A)** Zn-AL and **(B)** Zn-ST.

Figure 13 shows the UV-Vis absorbance spectra of ZnO samples before and after Cu (II) adsorption. A broad peak in the visible region centered at 720 nm can be observed after the adsorption of copper. This peak can be assigned to the Cu (II) d-d transition (Li et al., 2013), indicating, the adsorption of Cu (II) ions by the ZnO nanoparticles (Zn-ST and Zn-AL), verifying the XRD results.

## Conclusions

ZnO nanoparticles have been successfully synthesized by an eco-friendly procedure based on a polysaccharide, without using any surfactant, organic solvent and at low calcination temperature. The type of polysaccharide used as fuel influences on the morphology and optical property of the synthesized nanoparticles (Zn-AL and Zn-ST). Cu (II) adsorption tests showed a low experimental (q_max_) value, however saturation was not observed on both synthesized (route I and route II) ZnO adsorbents in the 4 h study period. For both samples (Zn-AL and Zn-ST) at a concentration of 40 mg L^−1^ of copper ions, there was a high removal value of R%>95% indicating that the synthesized nanoparticles have the potential to be used in the treatment of wastewater, especially in the removal of metal ions at low concentrations. The XRD analysis of the samples after Cu (II) adsorption indicates the formation of the Tenorite phase on the ZnO nanoparticles surface regardless of the pH used in the adsorption experiment, denoting the formation of a secondary phase in the ZnO structure. Accordingly, the two ZnO synthesis routes favor controlling the surface charge, phase, crystallite size, modulating solids for specific applications (photocatalysis, sensor, pigments).

## Data Availability Statement

The raw data supporting the conclusions of this article will be made available by the authors, without undue reservation.

## Author Contributions

JP performed the methodology, conceptualization, investigation, and wrote the manuscript with input from CB, FA, and SJ. CB, SA, and JP performed XPS measurement and analysis. AS-C and J-FC performed the SEM measurement. VT investigation and formal data analysis of XRD. SJ performed of adsorption tests and discussion. J-FC, CB, and FA supervision. VT, CB, and FA funding acquisition and project administration. All authors contributed to the article and approved the submitted version.

## Conflict of Interest

The authors declare that the research was conducted in the absence of any commercial or financial relationships that could be construed as a potential conflict of interest.
